# Supplementary material for: Genetic context drives age‐related disparities in synaptic maintenance and structure across cortical and hippocampal neuronal circuits
Source: Aging Cell. 2023 Dec 21;23(2):e14033. doi: 10.1111/acel.14033 (PMC10861192; doi:10.1111/acel.14033)
Supplement: Supplementary file 1 — Data S1. [file ACEL-23-e14033-s001.docx]

**SUPPLEMENTARY INFORMATION**

**Genetic context drives age-related disparities in synaptic maintenance and structure across cortical and hippocampal neuronal circuits.**

Sarah E. Heuer, Emily W. Nickerson, Gareth R. Howell, Erik B. Bloss

**
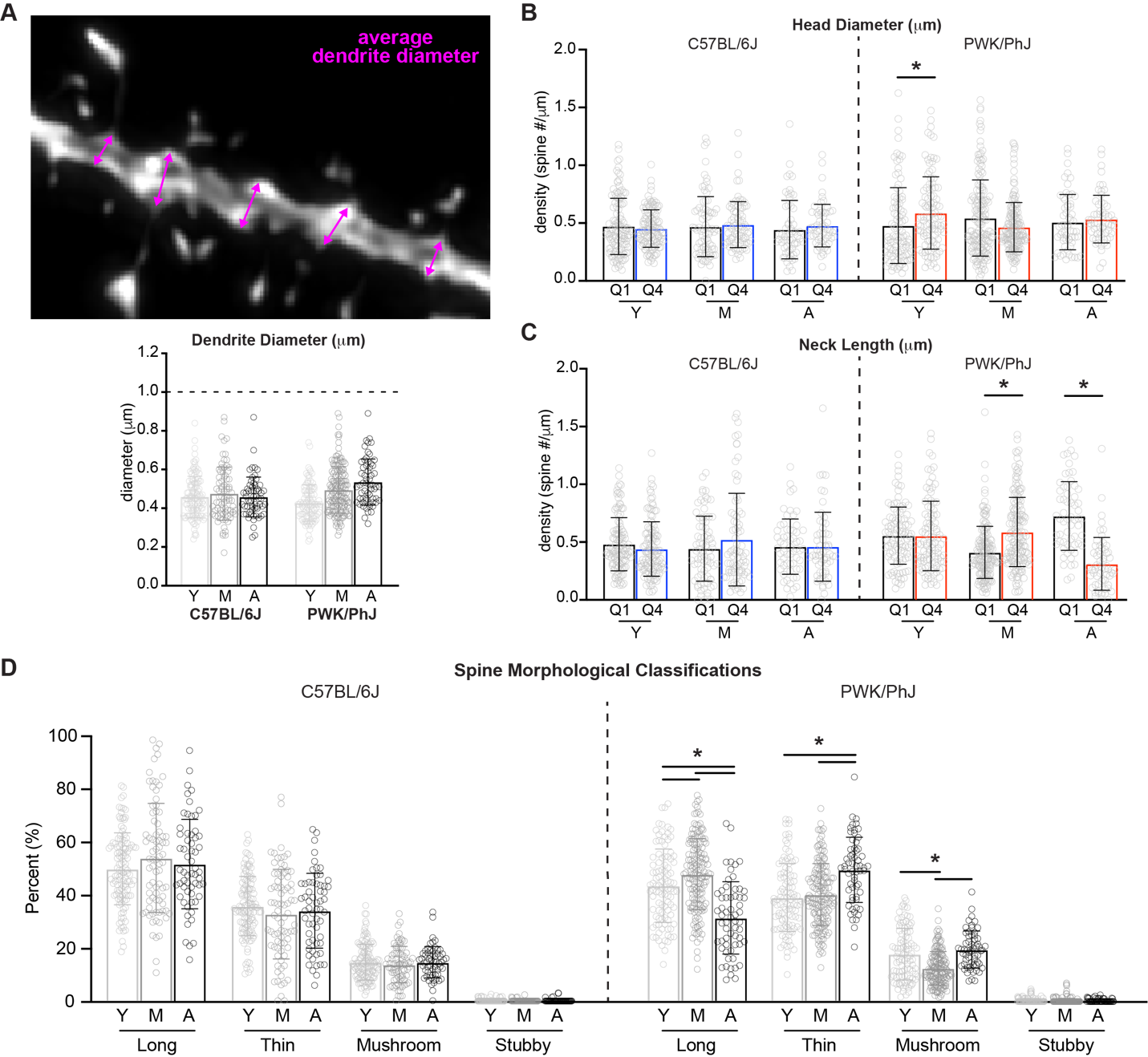
**

**Figure S1: Additional morphological analyses for proximal CA1-to-PFC spines and dendrites, corresponding to Figure 2.**

**(A)** CA1-to-PFC proximal average dendrite diameter example image (top) and quantification of average diameter for each analyzed dendrite (bottom). Horizontal dashed line represents that dendrites sampled were under 1 μm in diameter, corresponding to uniformly thin branches. Data points represent individual branches (n=20/mouse). Groups measured include young (Y), middle-aged (M) and aged (A).

**(B)** Quartile-based analyses of CA1-to-PFC proximal spine head diameters. All proximal CA1-to-PFC spines within each strain were divided into quartiles based on head diameter (μm). The smallest spines assigned to the first quartile (Q1) and the largest spines assigned to the fourth quartile (Q4) were identified and reassigned back to originating dendrite. Spine densities (spines/μm) for Q1 and Q4 spines were calculated separately. Data points represent individual branches. Nonparametric two-tailed t-tests were performed to compare Q1 to Q4 within each strain/age group to identify significant (*=p<0.05) shifts in size (see **Table S2**).

**(C)** Same as **(B)** for CA1-to-PFC proximal spine neck length.

**(D)** Spine morphological classifications. All spines belonging to proximal CA1-to-PFC dendrites were assigned a morphology grouping based on criteria outlined in *Methods*. The percent composition of each spine-type was calculated for each dendrite, and changes in spine-type composition compared across age-groups. Data point represent percent composition of each spine-type from individual branches. One-way ANOVA were performed followed by Bonferroni post-hoc pairwise analyses to determine significant (*=adj.p<0.05) effects of age on spine-type composition (see **Table S2**). Groups measured include young (Y), middle-aged (M) and aged (A).


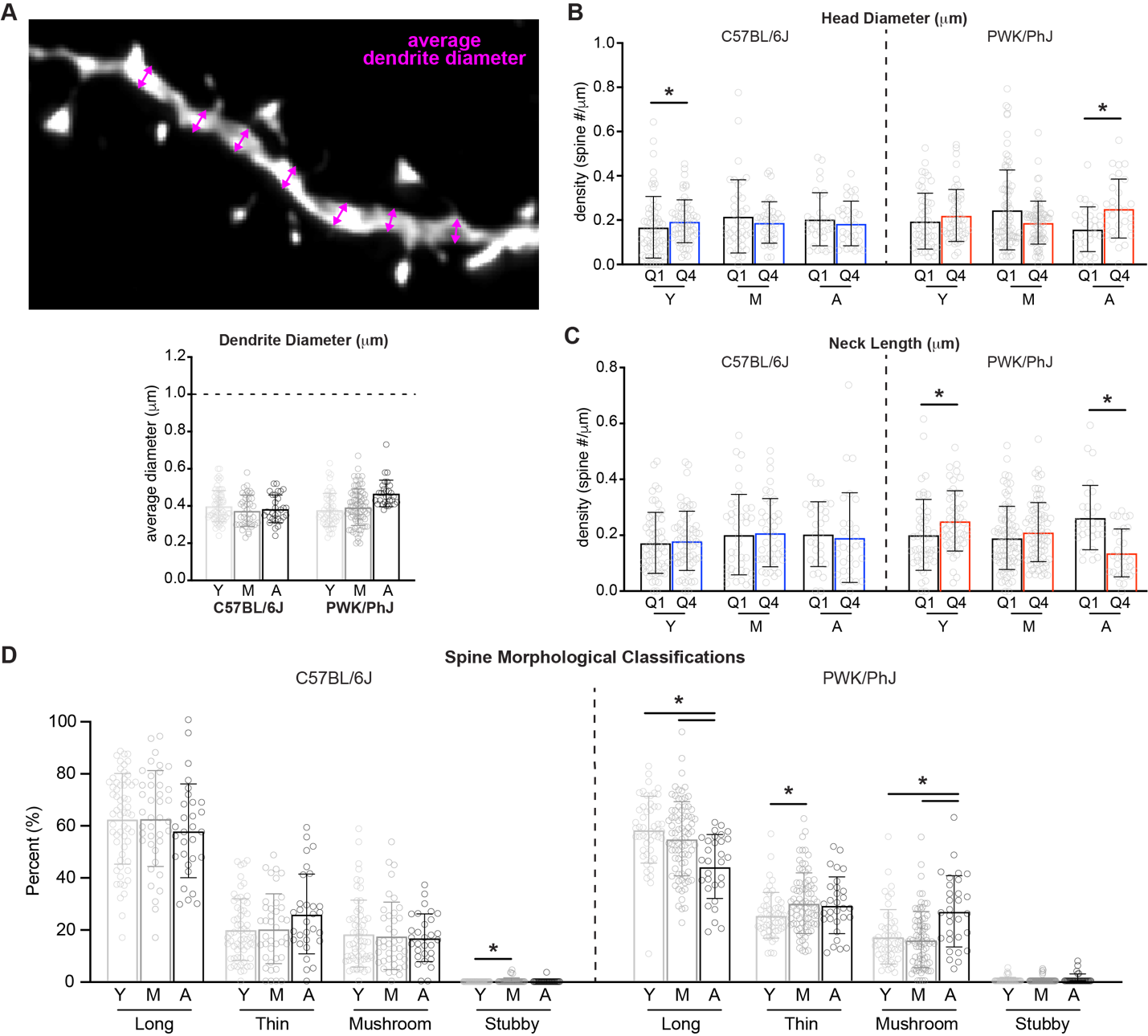


**Figure S2: Additional morphological analyses for distal tuft CA1-to-PFC spines and dendrites, corresponding to Figure 3.**

**(A)** CA1-to-PFC distal average dendrite diameter example image (top) and quantification of average diameter for each analyzed dendrite (bottom). Horizontal dashed line represents that dendrites sampled were under 1 μm in diameter, corresponding to uniformly thin branches. Data points represent individual branches (n=10/mouse). Groups measured include young (Y), middle-aged (M) and aged (A).

**(B)** Quartile-based analyses of distal tuft CA1-to-PFC spine head diameters. All distal tuft CA1-to-PFC spines within each strain were divided into quartiles based on head diameter (μm). The smallest spines assigned to the first quartile (Q1) and the largest spines assigned to the fourth quartile (Q4) were identified and reassigned back to originating dendrite. Spine densities (spines/μm) for Q1 and Q4 spines were calculated separately. Data points represent individual branches. Nonparametric two-tailed t-tests were performed to compare Q1 to Q4 within each strain/age group to identify significant (*=p<0.05) shifts in size (see **Table S3**).

**(C)** Same as **(B)** for CA1-to-PFC distal tuft spine neck length.

**(D)** Spine morphological classifications. All spines belonging to distal CA1-to-PFC dendrites were assigned a morphology grouping based on criteria outlined in *Methods*. The percent composition of each spine-type was calculated for each dendrite, and changes in spine-type composition compared across age-groups. Data point represent percent composition of each spine-type from individual branches. One-way ANOVA were performed followed by Bonferroni post-hoc pairwise analyses to determine significant (*=adj.p<0.05) effects of age on spine-type composition (see **Table S3**). Groups measured include young (Y), middle-aged (M) and aged (A).

**
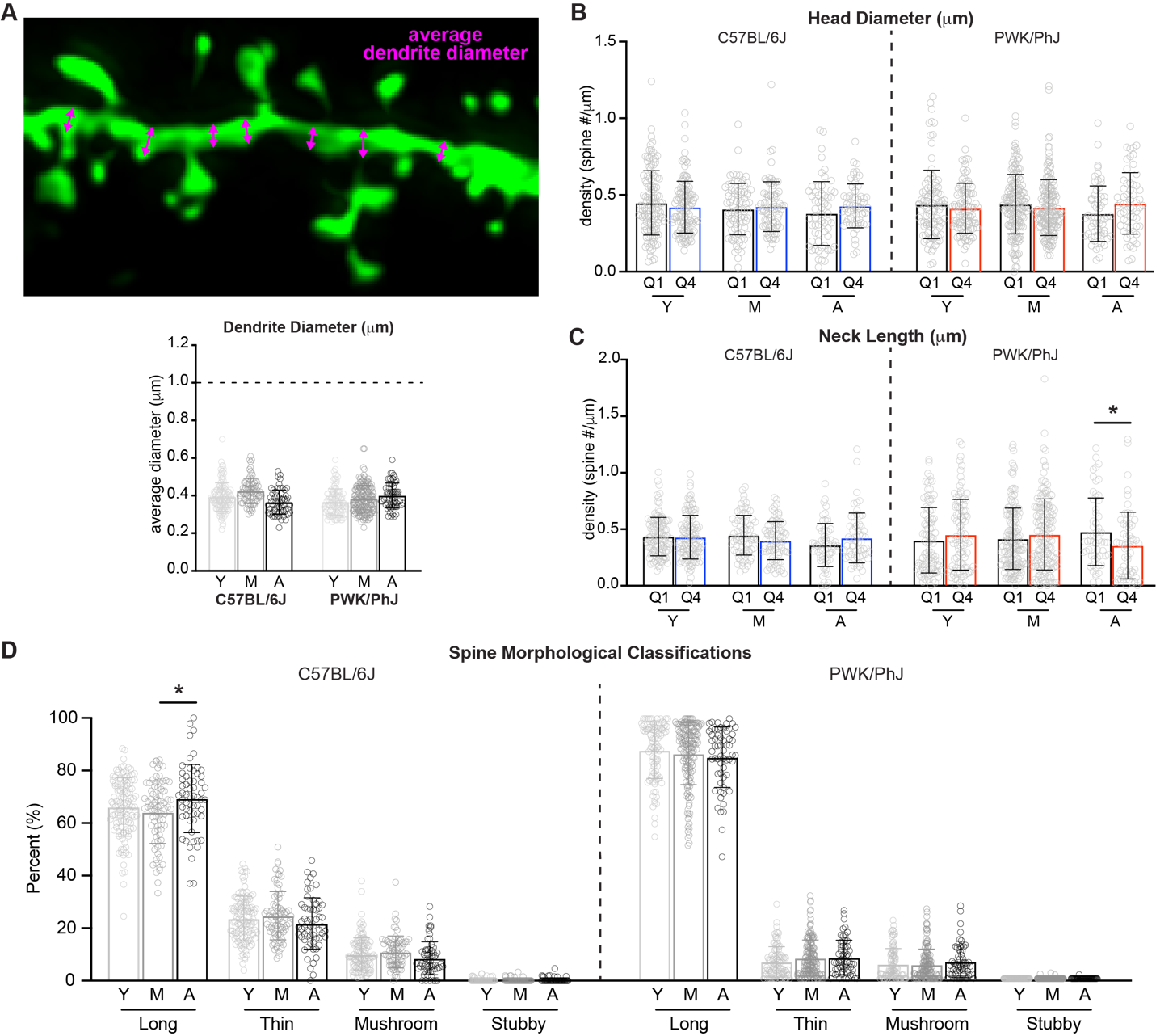
**

**Figure S3: Additional morphological analyses for distal tuft PFC-to-RE spines and dendrites, corresponding to Figure 4.**

**(A)** PFC-to-RE proximal average dendrite diameter example image (top) and quantification of average diameter for each analyzed dendrite (bottom). Horizontal dashed line represents that dendrites sampled were under 1 μm in diameter, corresponding to uniformly thin branches. Data points represent individual branches (n=20/mouse). Groups measured include young (Y), middle-aged (M) and aged (A).

**(B)** Quartile-based analyses of proximal PFC-to-RE spine head diameters. All proximal PFC-to-RE spines within each strain were divided into quartiles based on head diameter (μm). The smallest spines assigned to the first quartile (Q1) and the largest spines assigned to the fourth quartile (Q4) were identified and reassigned back to originating dendrite. Spine densities (spines/μm) for Q1 and Q4 spines were calculated separately. Data points represent individual branches. Nonparametric two-tailed t-tests were performed to compare Q1 to Q4 within each strain/age group to identify significant (*=p<0.05) shifts in size (see **Table S4**).

**(C)** Same as **(B)** for PFC-to-RE proximal spine neck length.

**(D)** Spine morphological classifications. All spines belonging to proximal PFC-to-RE dendrites were assigned a morphology grouping based on criteria outlined in *Methods*. The percent composition of each spine-type was calculated for each dendrite, and changes in spine-type composition compared across age-groups. Data point represent percent composition of each spine-type from individual branches. One-way ANOVA were performed followed by Bonferroni post-hoc pairwise analyses to determine significant (*=adj.p<0.05) effects of age on spine-type composition (see **Table S4**). Groups measured include young (Y), middle-aged (M) and aged (A).


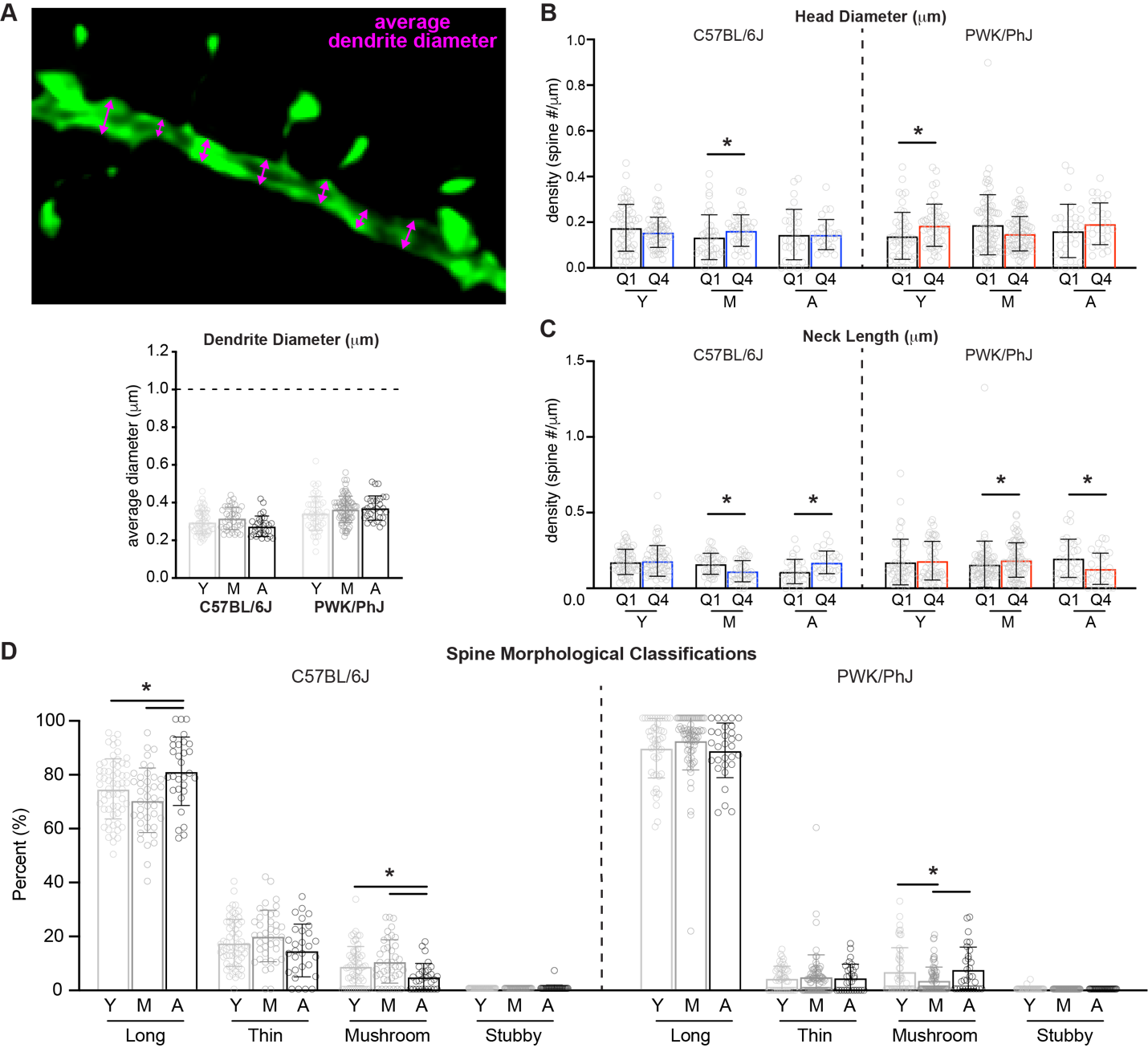


**Figure S4: Additional morphological analyses for distal tuft PFC-to-RE spines and dendrites, corresponding to Figure 5.**

**(A)** PFC-to-RE distal average dendrite diameter example image (top) and quantification of average diameter for each analyzed dendrite (bottom). Horizontal dashed line represents that dendrites sampled were under 1 μm in diameter, corresponding to uniformly thin branches. Data points represent individual branches (n=10/mouse). Groups measured include young (Y), middle-aged (M) and aged (A).

**(B)** Quartile-based analyses of distal tuft PFC-to-RE spine head diameters. All distal tuft PFC-to-RE spines within each strain were divided into quartiles based on head diameter (μm). The smallest spines assigned to the first quartile (Q1) and the largest spines assigned to the fourth quartile (Q4) were identified and reassigned back to originating dendrite. Spine densities (spines/μm) for Q1 and Q4 spines were calculated separately. Data points represent individual branches. Nonparametric two-tailed t-tests were performed to compare Q1 to Q4 within each strain/age group to identify significant (*=p<0.05) shifts in size (see **Table S5**).

**(C)** Same as **(B)** for PFC-to-RE distal tuft spine neck length.

**(D)** Spine morphological classifications. All spines belonging to distal PFC-to-RE dendrites were assigned a morphology grouping based on criteria outlined in *Methods*. The percent composition of each spine-type was calculated for each dendrite, and changes in spine-type composition compared across age-groups. Data point represent percent composition of each spine-type from individual branches. One-way ANOVA were performed followed by Bonferroni post-hoc pairwise analyses to determine significant (*=adj.p<0.05) effects of age on spine-type composition (see **Table S5**). Groups measured include young (Y), middle-aged (M) and aged (A).­­­


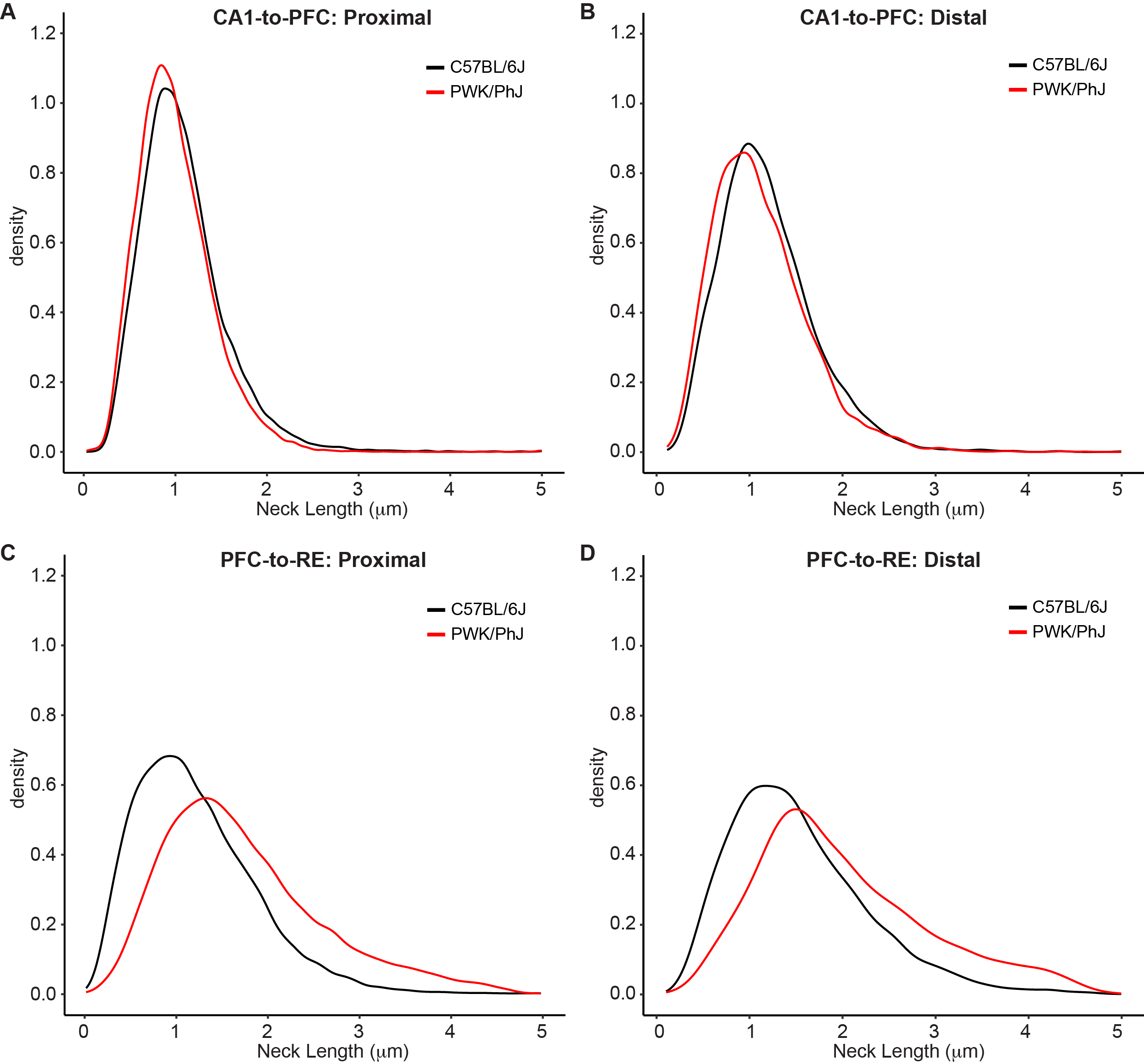


**Figure S5: Distributions of spine neck lengths between C57BL/6J and PWK/PhJ mice show PFC-to-RE spines from PWK/PhJ are longer than C57BL/6J.**

**(A)** Distribution of spine neck lengths in CA1-to-PFC proximal dendrites from C57BL/6J (black) and PWK/PhJ (red) mice. All data were collapsed by age, with data representing total spine population from examined dendrites.

**(B)** Same as (A) for distal CA1-to-PFC dendrites.

**(C)** Same as (A) for proximal PFC-to-RE dendrites.

**(D)** Same as (A) for distal PFC-to-RE dendrites.

**SUPPLEMENTAL TABLES**

**Table S1- Mouse information for current study, corresponding to Figure 1**

| **MouseID** | **Strain** | **Sex** | **DOB** | **Harvest Date** | **Age (m)** | **Age Group** | **Terminal body weight (g)** |
| --- | --- | --- | --- | --- | --- | --- | --- |
| 59944 | PWK/PhJ | F | 11/24/2019 | 5/21/2022 | 30 | Aged | 18.4 |
| 59246 | PWK/PhJ | F | 4/24/2020 | 5/21/2022 | 25 | Aged | 24.6 |
| 59247 | PWK/PhJ | F | 4/24/2020 | 5/21/2022 | 25 | Aged | 23.3 |
| 76266 | PWK/PhJ | F | 3/9/2021 | 8/8/2022 | 17 | Middle-Aged | 17.1 |
| 76267 | PWK/PhJ | F | 3/9/2021 | 8/8/2022 | 17 | Middle-Aged | 21.6 |
| 60938 | PWK/PhJ | F | 3/16/2021 | 6/13/2022 | 15 | Middle-Aged | 19.4 |
| 60939 | PWK/PhJ | F | 3/16/2021 | 6/13/2022 | 15 | Middle-Aged | 23.9 |
| 60942 | PWK/PhJ | F | 3/16/2021 | 6/13/2022 | 15 | Middle-Aged | 18.1 |
| 56621 | PWK/PhJ | F | 6/23/2021 | 6/30/2022 | 12 | Middle-Aged | 21.1 |
| 56622 | PWK/PhJ | F | 6/23/2021 | 6/30/2022 | 12 | Middle-Aged | 18.6 |
| 56833 | PWK/PhJ | F | 7/14/2021 | 6/30/2022 | 11 | Middle-Aged | 17.2 |
| 70328 | PWK/PhJ | F | 11/5/2021 | 6/13/2022 | 7 | Young | 16.3 |
| 70329 | PWK/PhJ | F | 11/5/2021 | 6/13/2022 | 7 | Young | 18.0 |
| 27811R | PWK/PhJ | F | 1/11/2022 | 6/13/2022 | 5 | Young | 16.1 |
| 2781B | PWK/PhJ | F | 1/11/2022 | 6/13/2022 | 5 | Young | 17.6 |
| 27812L | PWK/PhJ | F | 1/11/2022 | 6/13/2022 | 5 | Young | 16.9 |
| 2430A | C57BL/6J | F | 8/9/2022 | 12/13/2022 | 4 | Young | 21.1 |
| 2430B | C57BL/6J | F | 8/9/2022 | 12/13/2022 | 4 | Young | 21.0 |
| A0003 | C57BL/6J | F | 5/31/2022 | 11/4/2022 | 6 | Young | 25.7 |
| A0004 | C57BL/6J | F | 5/31/2022 | 11/4/2022 | 6 | Young | 23.8 |
| 24311R | C57BL/6J | F | 5/10/2022 | 12/18/2022 | 7 | Young | 23.9 |
| 2432A | C57BL/6J | F | 5/10/2022 | 12/18/2022 | 7 | Young | 27.6 |
| 5551R | C57BL/6J | F | 11/23/2021 | 12/18/2022 | 13 | Middle-Aged | 25.3 |
| 555B | C57BL/6J | F | 11/23/2021 | 12/13/2022 | 13 | Middle-Aged | 26.1 |
| 15721R | C57BL/6J | F | 7/13/2021 | 12/13/2022 | 17 | Middle-Aged | 27.4 |
| 15722R | C57BL/6J | F | 7/13/2021 | 12/18/2022 | 17 | Middle-Aged | 36.9 |
| 60840 | C57BL/6J | F | 2/16/2021 | 12/13/2022 | 22 | Aged | 35.7 |
| A3924 | C57BL/6J | F | 12/2/2020 | 11/4/2022 | 23 | Aged | 37.9 |
| A3926 | C57BL/6J | F | 12/2/2020 | 12/18/2022 | 24 | Aged | 29.1 |

**Table S2- Associated statistics for proximal CA1 dendrites, corresponding to Figure 2 and Figure S1**

**S2A. Summary statistics for proximal CA1-to-PFC spine data**

| **Strain** | **Age Group** | **Mouse N** | **Dendrite N** | **Spine N** |
| --- | --- | --- | --- | --- |
| B6 | Young | 6 | 120 | 7479 |
| B6 | Middle-Aged | 4 | 80 | 4845 |
| B6 | Aged | 3 | 60 | 3611 |
| PWK | Young | 5 | 100 | 7125 |
| PWK | Middle-Aged | 8 | 160 | 10882 |
| PWK | Aged | 3 | 60 | 4494 |

**S2B. Spine density ANOVA followed by Bonferroni post-hoc pairwise analysis**

| **Group A** | **Group B** | **C57BL/6J**  **Adj. p-value** | **PWK/PhJ**  **Adj. p-value** |
| --- | --- | --- | --- |
| Young | Middle-Aged | >0.9999 | >0.9999 |
| Middle-Aged | Aged | 0.9088 | 0.6156 |
| Young | Aged | 0.2297 | >0.9999 |
| One-way ANOVA | | F = 1.584  p-value = 0.2071 | F = 0.8171  p-value = 0.4427 |

**S2C. Head Diameter Kolmogorov-Smirnov tests**

|  |  | **C57BL/6J** | | **PWK/PhJ** | |
| --- | --- | --- | --- | --- | --- |
| **Group A** | **Group B** | **KS p-value** | **K-S Bonferroni adjusted pvalue** | **KS p-value** | **K-S Bonferroni adjusted pvalue** |
| Young | Middle-Aged | 0.02206 | 0.06618 | 2.2e-16 | 6.6e-16 |
| Middle-Aged | Aged | 0.07032 | 0.21096 | 0.006142 | 0.018426 |
| Young | Aged | 0.01216 | 0.03648 | 3.542e-07 | 1.0626E-06 |

**S2D. Head diameter quartile nonparametric t-tests**

| **Group A** | **Group B** | **C57BL/6J**  **p-value** | **PWK/PhJ**  **p-value** |
| --- | --- | --- | --- |
| Young Q1 | Young Q4 | 0.8133 | 0.0030 |
| Middle-Aged Q1 | Middle-Aged Q4 | 0.2422 | 0.1786 |
| Aged Q1 | Aged Q4 | 0.0715 | 0.3116 |

**S2E. Neck Length Kolmogorov-Smirnov tests**

|  |  | **C57BL/6J** | | **PWK/PhJ** | |
| --- | --- | --- | --- | --- | --- |
| **Group A** | **Group B** | **KS p-value** | **K-S Bonferroni adjusted pvalue** | **KS p-value** | **K-S Bonferroni adjusted pvalue** |
| Young | Middle-Aged | 4.836e-11 | 1.4508E-10 | 2.2e-16 | 6.6e-16 |
| Middle-Aged | Aged | 0.002058 | 0.006174 | 2.2e-16 | 6.6e-16 |
| Young | Aged | 0.02071 | 0.06213 | 2.2e-16 | 6.6e-16 |

**S2F. Neck length quartile nonparametric t-tests**

| **Group A** | **Group B** | **C57BL/6J**  **p-value** | **PWK/PhJ**  **p-value** |
| --- | --- | --- | --- |
| Young Q1 | Young Q4 | 0.1029 | 0.4625 |
| Middle-Aged Q1 | Middle-Aged Q4 | 0.6502 | <0.0001 |
| Aged Q1 | Aged Q4 | 0.5712 | <0.0001 |

**S2G. Spine classification ANOVA followed by Bonferroni correction**

| **Spine Type** | **Group A** | **Group B** | **C57BL/6J**  **Adj. p-value** | **PWK/PhJ**  **Adj. p-value** |
| --- | --- | --- | --- | --- |
| Long | Young | Middle-Aged | 0.2794 | 0.0362 |
|  | Middle-Aged | Aged | >0.9999 | <0.0001 |
|  | Young | Aged | >0.9999 | <0.0001 |
|  | One-way ANOVA | | F = 1.422  p-value = 0.2430 | F = 32.01  p-value = <0.0001 |
| Thin | Young | Middle-Aged | 0.3913 | >0.9999 |
|  | Middle-Aged | Aged | >0.9999 | <0.0001 |
|  | Young | Aged | >0.9999 | <0.0001 |
|  | One-way ANOVA | | F = 1.186  p-value = 0.3072 | F = 15.98  p-value = <0.0001 |
| Mushroom | Young | Middle-Aged | 0.9187 | <0.0001 |
|  | Middle-Aged | Aged | >0.9999 | <0.0001 |
|  | Young | Aged | >0.9999 | 0.5284 |
|  | One-way ANOVA | | F = 0.5867  p-value = 0.5569 | F = 25.55  p-value = <0.0001 |
| Stubby | Young | Middle-Aged | >0.9999 | 0.7165 |
|  | Middle-Aged | Aged | >0.9999 | >0.9999 |
|  | Young | Aged | >0.9999 | >0.9999 |
|  | One-way ANOVA | | F = 0.5005  p-value = 0.6068 | F = 0.7015  p-value = 0.4966 |

**Table S3- Associated statistics for distal tuft CA1 dendrites, corresponding to Figure 3 and Figure S2**

**S3A. Summary statistics for distal CA1-to-PFC spine data**

| **Strain** | **Age Group** | **Mouse N** | **Dendrite N** | **Spine N** |
| --- | --- | --- | --- | --- |
| B6 | Young | 6 | 60 | 1370 |
| B6 | Middle-Aged | 4 | 40 | 1003 |
| B6 | Aged | 3 | 30 | 700 |
| PWK | Young | 5 | 50 | 1512 |
| PWK | Middle-Aged | 8 | 80 | 2123 |
| PWK | Aged | 3 | 30 | 849 |

**S3B. Spine density ANOVA followed by Bonferroni post-hoc pairwise analysis**

| **Group A** | **Group B** | **C57BL/6J**  **Adj. p-value** | **PWK/PhJ**  **Adj. p-value** |
| --- | --- | --- | --- |
| Young | Middle-Aged | >0.9999 | >0.9999 |
| Middle-Aged | Aged | >0.9999 | >0.9999 |
| Young | Aged | >0.9999 | >0.9999 |
| One-way ANOVA | | F = 0.4320  p-value = 0.6502 | F = 0.2171  p-value = 0.8051 |

**S3C. Head Diameter Kolmogorov-Smirnov tests**

|  |  | **C57BL/6J** | | **PWK/PhJ** | |
| --- | --- | --- | --- | --- | --- |
| **Group A** | **Group B** | **KS p-value** | **K-S Bonferroni adjusted pvalue** | **KS p-value** | **K-S Bonferroni adjusted pvalue** |
| Young | Middle-Aged | 0.004019 | 0.012057 | 0.0004517 | 0.0013551 |
| Middle-Aged | Aged | 0.1044 | 0.3132 | 4.668e-09 | 1.4004E-08 |
| Young | Aged | 0.2682 | 0.8046 | 0.005925 | 0.017775 |

**S3D. Head diameter quartile nonparametric t-tests**

| **Group A** | **Group B** | **C57BL/6J**  **p-value** | **PWK/PhJ**  **p-value** |
| --- | --- | --- | --- |
| Young Q1 | Young Q4 | 0.0382 | 0.1527 |
| Middle-Aged Q1 | Middle-Aged Q4 | 0.9268 | 0.2656 |
| Aged Q1 | Aged Q4 | 0.7330 | 0.0060 |

**S3E. Neck Length Kolmogorov-Smirnov tests**

|  |  | **C57BL/6J** | | **PWK/PhJ** | |
| --- | --- | --- | --- | --- | --- |
| **Group A** | **Group B** | **KS p-value** | **K-S Bonferroni adjusted pvalue** | **KS p-value** | **KS Bonferroni adjusted pvalue** |
| Young | Middle-Aged | 0.03513 | 0.10539 | 0.06974 | 0.20922 |
| Middle-Aged | Aged | 0.2409 | 0.7227 | 9.98e-07 | 2.994E-06 |
| Young | Aged | 0.01058 | 0.03174 | 2.762e-08 | 8.286E-08 |

**S3F. Neck length quartile nonparametric t-tests**

| **Group A** | **Group B** | **C57BL/6J**  **p-value** | **PWK/PhJ**  **p-value** |
| --- | --- | --- | --- |
| Young Q1 | Young Q4 | 0.5801 | 0.0089 |
| Middle-Aged Q1 | Middle-Aged Q4 | 0.6170 | 0.1903 |
| Aged Q1 | Aged Q4 | 0.3561 | <0.0001 |

**S3G. Spine classification ANOVA followed by Bonferroni correction**

| **Spine Type** | **Group A** | **Group B** | **C57BL/6J**  **Adj. p-value** | **PWK/PhJ**  **Adj. p-value** |
| --- | --- | --- | --- | --- |
| Long | Young | Middle-Aged | >0.9999 | 0.4607 |
|  | Middle-Aged | Aged | 0.8342 | 0.0009 |
|  | Young | Aged | 0.7683 | <0.0001 |
|  | One-way ANOVA | | F = 0.7714  p-value = 0.4645 | F = 10.67  p-value = <0.0001 |
| Thin | Young | Middle-Aged | >0.9999 | 0.0497 |
|  | Middle-Aged | Aged | 0.2333 | >0.9999 |
|  | Young | Aged | 0.1312 | 0.3588 |
|  | One-way ANOVA | | F = 2.294  p-value = 0.1051 | F = 3.038  p-value = 0.0507 |
| Mushroom | Young | Middle-Aged | >0.9999 | >0.9999 |
|  | Middle-Aged | Aged | >0.9999 | <0.0001 |
|  | Young | Aged | >0.9999 | 0.0007 |
|  | One-way ANOVA | | F = 0.1768  p-value = 0.8382 | F = 10.71  p-value = <0.0001 |
| Stubby | Young | Middle-Aged | 0.0341 | >0.9999 |
|  | Middle-Aged | Aged | 0.4387 | 0.1176 |
|  | Young | Aged | >09999 | 0.3671 |
|  | One-way ANOVA | | F = 3.317  p-value = 0.0394 | F = 2.183  p-value = 0.1161 |

**Table S4- Associated statistics for proximal PFC dendrites, corresponding to Figure 4 and Figure S3**

**S4A. Summary statistics for proximal PFC-to-RE spine data**

| **Strain** | **Age Group** | **Mouse N** | **Dendrite N** | **Spine N** |
| --- | --- | --- | --- | --- |
| B6 | Young | 6 | 120 | 7997 |
| B6 | Middle-Aged | 4 | 80 | 5126 |
| B6 | Aged | 3 | 60 | 3419 |
| PWK | Young | 5 | 100 | 6754 |
| PWK | Middle-Aged | 8 | 160 | 10611 |
| PWK | Aged | 3 | 60 | 3853 |

**S4B. Spine density ANOVA followed by Bonferroni post-hoc pairwise analysis**

| **Group A** | **Group B** | **C57BL/6J**  **Adj. p-value** | **PWK/PhJ**  **Adj. p-value** |
| --- | --- | --- | --- |
| Young | Middle-Aged | 0.0339 | >0.9999 |
| Middle-Aged | Aged | 0.1552 | 0.4328 |
| Young | Aged | <0.0001 | 0.1846 |
| One-way ANOVA | | F = 10.36  p-value < 0.0001 | F = 1.796  p-value = 0.1676 |

**S4C. Head Diameter Kolmogorov-Smirnov tests**

|  |  | **C57BL/6J** | | **PWK/PhJ** | |
| --- | --- | --- | --- | --- | --- |
| **Group A** | **Group B** | **KS p-value** | **K-S Bonferroni adjusted pvalue** | **KS p-value** | **K-S Bonferroni adjusted pvalue** |
| Young | Middle-Aged | 0.003496 | 0.010488 | 0.3364 | 1 |
| Middle-Aged | Aged | 0.02801 | 0.08403 | 0.003807 | 0.011421 |
| Young | Aged | 7.682e-05 | 0.00023046 | 0.00315 | 0.00945 |

**S4D. Head Diameter quartile nonparametric t-tests**

| **Group A** | **Group B** | **C57BL/6J**  **p-value** | **PWK/PhJ**  **p-value** |
| --- | --- | --- | --- |
| Young Q1 | Young Q4 | 0.3153 | 0.7610 |
| Middle-Aged Q1 | Middle-Aged Q4 | 0.8602 | 0.1076 |
| Aged Q1 | Aged Q4 | 0.1300 | 0.0503 |

**S4E. Neck Length Kolmogorov-Smirnov tests**

|  |  | **C57BL/6J** | | **PWK/PhJ** | |
| --- | --- | --- | --- | --- | --- |
| **Group A** | **Group B** | **KS p-value** | **K-S Bonferroni adjusted pvalue** | **KS p-value** | **K-S Bonferroni adjusted pvalue** |
| Young | Middle-Aged | 0.01101 | 0.03303 | 0.03866 | 0.11598 |
| Middle-Aged | Aged | 1.002e-07 | 3.006e-07 | 5.029e-14 | 1.5087e-13 |
| Young | Aged | 0.0009113 | 0.0027339 | 2.343e-14 | 7.029e-14 |

**S4F. Neck length quartile nonparametric t-tests**

| **Group A** | **Group B** | **C57BL/6J**  **p-value** | **PWK/PhJ**  **p-value** |
| --- | --- | --- | --- |
| Young Q1 | Young Q4 | 0.6594 | 0.3307 |
| Middle-Aged Q1 | Middle-Aged Q4 | 0.1251 | 0.3613 |
| Aged Q1 | Aged Q4 | 0.1942 | 0.0179 |

**S4G. Spine classification ANOVA followed by Bonferroni correction**

| **Spine Type** | **Group A** | **Group B** | **C57BL/6J**  **Adj. p-value** | **PWK/PhJ**  **Adj. p-value** |
| --- | --- | --- | --- | --- |
| Long | Young | Middle-Aged | 0.7443 | >0.9999 |
|  | Middle-Aged | Aged | 0.0314 | >0.9999 |
|  | Young | Aged | 0.2552 | 0.4545 |
|  | One-way ANOVA | | F = 3.340  p-value = 0.0370 | F = 1.067  p-value = 0.3451 |
| Thin | Young | Middle-Aged | >0.9999 | 0.2168 |
|  | Middle-Aged | Aged | 0.1669 | 0.3442 |
|  | Young | Aged | 0.5574 | >0.9999 |
|  | One-way ANOVA | | F = 1.864  p-value = 0.1572 | F = 1.959  p-value = 0.1427 |
| Mushroom | Young | Middle-Aged | 0.6924 | >0.9999 |
|  | Middle-Aged | Aged | 0.0769 | 0.5851 |
|  | Young | Aged | 0.8167 | 0.9360 |
|  | One-way ANOVA | | F = 2.545  p-value = 0.0808 | F = 0.8604  p-value = 0.4240 |
| Stubby | Young | Middle-Aged | >0.9999 | 0.5255 |
|  | Middle-Aged | Aged | 0.5889 | >0.9999 |
|  | Young | Aged | 0.3897 | >0.9999 |
|  | One-way ANOVA | | F = 1.258  p-value = 0.2861 | F = 0.9344  p-value = 0.3939 |

**Table S5- Associated statistics for distal tuft PFC dendrites, corresponding to Figure 5 and Figure S4**

**S5A. Summary statistics for distal PFC-to-RE spine data**

| **Strain** | **Age Group** | **Mouse N** | **Dendrite N** | **Spine N** |
| --- | --- | --- | --- | --- |
| B6 | Young | 6 | 60 | 1481 |
| B6 | Middle-Aged | 4 | 40 | 815 |
| B6 | Aged | 3 | 30 | 631 |
| PWK | Young | 5 | 50 | 1321 |
| PWK | Middle-Aged | 8 | 80 | 2085 |
| PWK | Aged | 3 | 30 | 740 |

**S5B. Spine density ANOVA followed by Bonferroni post-hoc pairwise analysis**

| **Group A** | **Group B** | **C57BL/6J**  **Adj. p-value** | **PWK/PhJ**  **Adj. p-value** |
| --- | --- | --- | --- |
| Young | Middle-Aged | 0.0001 | >0.9999 |
| Middle-Aged | Aged | >0.9999 | >0.9999 |
| Young | Aged | 0.0124 | 0.9075 |
| One-way ANOVA | | F = 9.963  p-value <0.0001 | F = 0.5352  p-value = 0.5866 |

**S5C. Head Diameter Kolmogorov-Smirnov tests**

|  |  | **C57BL/6J** | | **PWK/PhJ** | |
| --- | --- | --- | --- | --- | --- |
| **Group A** | **Group B** | **KS p-value** | **K-S Bonferroni adjusted pvalue** | **KS p-value** | **K-S Bonferroni adjusted pvalue** |
| Young | Middle-Aged | 0.001061 | 0.003183 | 2.009e-06 | 6.027e-06 |
| Middle-Aged | Aged | 0.008744 | 0.026232 | 4.142e-05 | 0.00012426 |
| Young | Aged | 0.1408 | 0.4224 | 0.07482 | 0.22446 |

**S5D. Head diameter quartile nonparametric t-tests**

| **Group A** | **Group B** | **C57BL/6J**  **p-value** | **PWK/PhJ**  **p-value** |
| --- | --- | --- | --- |
| Young Q1 | Young Q4 | 0.3441 | 0.0073 |
| Middle-Aged Q1 | Middle-Aged Q4 | 0.0187 | 0.0944 |
| Aged Q1 | Aged Q4 | 0.8058 | 0.2065 |

**S5E. Neck Length Kolmogorov-Smirnov tests**

|  |  | **C57BL/6J** | | **PWK/PhJ** | |
| --- | --- | --- | --- | --- | --- |
| **Group A** | **Group B** | **KS p-value** | **K-S Bonferroni adjusted pvalue** | **KS p-value** | **K-S Bonferroni adjusted pvalue** |
| Young | Middle-Aged | 0.01932 | 0.05796 | 0.09178 | 0.27534 |
| Middle-Aged | Aged | 0.0003468 | 0.0010404 | 3.574e-05 | 0.00010722 |
| Young | Aged | 0.0131 | 0.0393 | 0.0006769 | 0.0020307 |

**S5F. Neck length quartile nonparametric t-tests**

| **Group A** | **Group B** | **C57BL/6J**  **p-value** | **PWK/PhJ**  **p-value** |
| --- | --- | --- | --- |
| Young Q1 | Young Q4 | 0.9760 | 0.6223 |
| Middle-Aged Q1 | Middle-Aged Q4 | 0.0023 | 0.0375 |
| Aged Q1 | Aged Q4 | 0.0019 | 0.0418 |

**S5G. Spine classification ANOVA followed by Bonferroni correction**

| **Spine Type** | **Group A** | **Group B** | **C57BL/6J**  **Adj. p-value** | **PWK/PhJ**  **Adj. p-value** |
| --- | --- | --- | --- | --- |
| Long | Young | Middle-Aged | 0.2404 | 0.4355 |
|  | Middle-Aged | Aged | 0.0007 | 0.3365 |
|  | Young | Aged | 0.0431 | >0.9999 |
|  | One-way ANOVA | | F = 7.194  p-value = 0.0011 | F = 1.777  p-value = 0.1726 |
| Thin | Young | Middle-Aged | 0.5576 | >0.9999 |
|  | Middle-Aged | Aged | 0.0533 | >0.9999 |
|  | Young | Aged | 0.5102 | >0.9999 |
|  | One-way ANOVA | | F = 2.892  p-value = 0.0591 | F = 0.1062  p-value = 0.8993 |
| Mushroom | Young | Middle-Aged | 0.7050 | 0.0300 |
|  | Middle-Aged | Aged | 0.0039 | 0.0218 |
|  | Young | Aged | 0.0456 | >0.9999 |
|  | One-way ANOVA | | F = 5.553  p-value = 0.0049 | F = 5.364  p-value = 0.0056 |
| Stubby | Young | Middle-Aged | >0.9999 | 0.1706 |
|  | Middle-Aged | Aged | 0.3485 | >0.9999 |
|  | Young | Aged | 0.2699 | 0.4087 |
|  | One-way ANOVA | | F = 1.684  p-value = 0.1897 | F = 2.056  p-value = 0.1314 |

**Table S6- Summary of spine responses to age within each strain**

**S6A. CA1 Spine changes: C57BL/6J**

|  |  | **Experimental measures** | | | **Predicted measures** | |
| --- | --- | --- | --- | --- | --- | --- |
|  | **Age Groups** | **Density** | **Head Diameter** | **Neck Length** | **EPSP spine** | **EPSP dendrite** |
| **Proximal** | Young 🡪 Middle-Aged | -- | -- | ↑ | -- | ↓ |
|  | Middle-Aged 🡪 Aged | -- | -- | ↓ | -- | ↑ |
|  | Young 🡪 Aged | -- | ↑ | -- | ↑ | -- |
| **Distal** | Young 🡪 Middle-Aged | -- | ↓ | -- | ↓ | -- |
|  | Middle-Aged 🡪 Aged | -- | -- | -- | -- | -- |
|  | Young 🡪 Aged | -- | -- | ↓ | -- | ↑ |

**S6B. CA1 Spine changes: PWK/PhJ**

|  |  | **Experimental measures** | | | **Predicted measures** | |
| --- | --- | --- | --- | --- | --- | --- |
|  | **Age Groups** | **Density** | **Head Diameter** | **Neck Length** | **EPSP spine** | **EPSP dendrite** |
| **Proximal** | Young 🡪 Middle-Aged | -- | ↓ | ↑ | ↓ | ↓ |
|  | Middle-Aged 🡪 Aged | -- | ↑ | ↓ | ↑ | ↑ |
|  | Young 🡪 Aged | -- | ↓ | ↓ | ↓ | ↑ |
| **Distal** | Young 🡪 Middle-Aged | -- | ↓ | -- | ↓ | -- |
|  | Middle-Aged 🡪 Aged | -- | ↑ | ↓ | ↑ | ↑ |
|  | Young 🡪 Aged | -- | ↑ | ↓ | ↑ | ↑ |

**S6C. PFC Spine changes: C57BL/6J**

|  |  | **Experimental measures** | | | **Predicted measures** | |
| --- | --- | --- | --- | --- | --- | --- |
|  | **Age Groups** | **Density** | **Head Diameter** | **Neck Length** | **EPSP spine** | **EPSP dendrite** |
| **Proximal** | Young 🡪 Middle-Aged | ↓ | ↑ | ↓ | ↑ | ↑ |
|  | Middle-Aged 🡪 Aged | -- | -- | ↑ | -- | ↓ |
|  | Young 🡪 Aged | ↓ | ↑ | ↑ | ↑ | ↓ |
| **Distal** | Young 🡪 Middle-Aged | ↓ | ↑ | -- | ↑ | -- |
|  | Middle-Aged 🡪 Aged | -- | ↓ | ↑ | ↓ | ↓ |
|  | Young 🡪 Aged | ↓ | -- | ↑ | -- | ↓ |

**S6D. PFC Spine changes: PWK/PhJ**

|  |  | **Experimental measures** | | | **Predicted measures** | |
| --- | --- | --- | --- | --- | --- | --- |
|  | **Age Groups** | **Density** | **Head Diameter** | **Neck Length** | **EPSP spine** | **EPSP dendrite** |
| **Proximal** | Young 🡪 Middle-Aged | -- | -- | -- | -- | -- |
|  | Middle-Aged 🡪 Aged | -- | ↑ | ↓ | ↑ | ↑ |
|  | Young 🡪 Aged | -- | ↑ | ↓ | ↑ | ↑ |
| **Distal** | Young 🡪 Middle-Aged | -- | ↓ | -- | ↓ | -- |
|  | Middle-Aged 🡪 Aged | -- | ↑ | ↓ | ↑ | ↑ |
|  | Young 🡪 Aged | -- | -- | ↓ | -- | ↑ |
